# Effect of Participation Motivation in Sports Climbing on Leisure Satisfaction and Physical Self-Efficacy

**DOI:** 10.3390/bs14010076

**Published:** 2024-01-22

**Authors:** Sheng Yen Lee, Sa Man Kim, Ryang Suk Lee, Ik Ryeul Park

**Affiliations:** 1 Department of Human Healthcare, Gyeongsang National University, Jinju-si 52725, Republic of Korea; 2 Graduate School of Social Physical Education, Korea National Sport University, Seoul 05541, Republic of Korea; 20190114@m365.knsu.ac.kr; 3 Graduate School of Hospitality and Tourism Management, Sejong University, Seoul 05006, Republic of Korea; lrs2552@naver.com

**Keywords:** motivation, leisure satisfaction, self-efficacy, physical activity, sports psychology, sports climbing

## Abstract

This study aimed to verify the effects of participation motivation in sports climbing on leisure satisfaction and physical self-efficacy. Structural equation modeling was used to analyze the causal relationships between participation motivation in sports climbing, leisure satisfaction, and physical self-efficacy, and to determine participation motivation. This study examined this causal relationship by verifying leisure satisfaction’s mediating effect on the relationship between participation motivation in sports climbing and physical self-efficacy. The participants of this study included 324 individuals over the age of 20 years with at least three months of sports climbing experience in the Seoul and Gyeonggi regions. The results indicated that among the subfactors of participation motivation in sports climbing, only skill acquisition and achievement positively affected leisure satisfaction, that leisure satisfaction positively affected physical self-efficacy, and that leisure satisfaction mediated the relationship between skill acquisition and achievement among the subfactors of participation motivation in sports climbing and physical self-efficacy. This study indicated that improved leisure satisfaction through sports climbing increases physical self-efficacy, including perceived improvement in physical abilities and confidence in interpersonal relationships. Accordingly, to expand and sustain participation, a systematic system for sports climbing instruction and educational programs is required to increase skill acquisition and a sense of accomplishment.

## 1. Introduction

### 1.1. Necessity for Research

As income and living standards improve, interest in health and quality of life increases, and leisure activity participation increases [1]. Traditionally, leisure consisted of hobbies and travel, centered on relaxation. However, as interest in health and quality of life increases due to economic growth and the development of medical technology, modern people have begun to recognize how to enjoy their leisure as an important factor in determining the quality of life [2]. This change in perception creates a social atmosphere that values sensibility and individuality, and interest in and participation in active leisure sports has increased [2,3].

Sports climbing can be divided into bouldering, lead climbing, and speed climbing and was previously considered the domain of trained experts [4] but began attracting the attention of young individuals and mountaineering enthusiasts seeking challenges and sensual leisure since its adoption as an official sport in the 2020 Olympic Games. The methods and characteristics of the three major sports climbing games, bouldering, lead climbing, and speed climbing, are as follows.

Bouldering is carried out on challenging routes, called “boulder problems”, on a low wall of 4.5 m without ropes, and rankings are determined based on the fewest attempts in a given period of time [5]. Lead climbing uses a rope, and the route is set on a 15 m wall. The ranking is determined by how high one climbs within the time limit on the same route installed in the arena [5]. Speed climbing is a game on a standard route on a 15 m-high wall with the same shape, position, and angle of holds. It is ranked based on completion time [5]. The demand for participation in sports climbing, which has been adopted as an Olympic sport as mentioned above, is rapidly increasing among non-athlete leisure participants [4]. Additionally, the number of professional indoor sports climbing gymnasiums has increased, spatial limitations and risks have been significantly reduced, and the interest base is greatly expanding [6].

Previous studies on sports and leisure, such as Ahn [2] and Kwak [4], suggested positive aspects of exercise. It has been reported that because various body parts are trained harmoniously, physical development and mental health are positively affected by relieving stress and increasing confidence [7]. Sports climbing has several positive aspects, including strengthening body muscles, improving endurance, and overcoming mental limitations through high-intensity physical activity [7,8]. In particular, sports climbing requires techniques with which to distribute body weight and conserve strength by using all parts of the body when climbing, as well as endurance to continue climbing. Besides advanced physical training that utilizes all parts of the body, sports climbing participants can improve creativity by finding and performing various types of climbing routes set in a climbing gym. It is a comprehensive sport that can improve abilities such as quickness and mental strength to find a different way in an instant when encountering difficulties [4]. However, it also contains relatively difficult elements compared to other leisure activities, such as risk factors due to the precarious nature of climbing and the physical strength and practice required to reach a certain level of skill [9].

Based on participation motivations in general sports activities, it is difficult to explain the current trend of increasing interest and participation in leisure activities such as sports climbing, which are called extreme sports due to their adventurous nature and risk factors and are enjoyed only by a small number of enthusiastic individuals. Although some previous studies have mentioned that participation motivation in sports climbing differs from that of other leisure sports [4,10], few have been conducted on the influence and effect of specific participation motivation.

Currently, limited research has been conducted on sports climbing. Previous studies have predominantly focused on exercise ability [11,12] and sports climbing facility operations [13,14]. Since the adoption of climbing as an official Olympic sport, the general public’s interest has increased, and the number of participants is increasing. Therefore, it is necessary to verify the various factors related to participation in sports climbing.

Compared to other leisure activities, sports climbing has characteristics that make it difficult to access, such as risk and difficulty; therefore, it is necessary to verify the reason for participation. A previous study, such as Liu and Ko [15], suggested that participation motivation varies in satisfaction and subsequent behavior for each motivation type.

Satisfaction is one of the most important indicators of participants’ leisure behavior, and some previous studies suggested that leisure activities that involve physical activity may have a higher impact on satisfaction than static leisure activities [16]. Participation in leisure sports involving physical activity has a strong psychological, physiological, and social impact, ultimately affecting quality of life [17]. Additionally, identifying self-efficacy through sports climbing activities not only allows for the analysis of participants’ exercise performance but also provides a detailed understanding of the exercise characteristics of sports climbing.

This study sought to verify the impact of participation motivation in sports climbing on leisure satisfaction and physical self-efficacy among sports climbers, on which little related research has been conducted. Specifically, this study analyzed the motivation to participate in sports climbing and set leisure satisfaction as a mediating factor to understand the perceived effects of exercise through a comprehensive analysis of participation motivation and physical self-efficacy. With our results, we aimed to identify the athletic and participation characteristics of sports climbing and provide basic data for continued participation in the sport and the expansion of its base.

### 1.2. Literature Review

#### 1.2.1. Participation Motivation

Participation motivation refers to the desire to connect inner intentions to actual external actions and plays a role in eliciting actions, such as learning and exercising [18].

Motivation is an important factor in sports activities involving physical movement because it can predict the reasons for participation, continuation, extent, and outcome. Ryan and Deci [19] classified participation motivation into three types: intrinsic, extrinsic, and amotivation. Intrinsic motivation is an expression of the voluntary will to achieve a personal goal or achievement, whereas extrinsic motivation is predominantly viewed as a reward that can be obtained when achieving a result or mandatory participation owing to recommendations and necessities from others. Regarding leisure, participation motivation appears to be strongly influenced by an inherent desire to escape daily life [20]. Additionally, previous studies have suggested that autonomous participants had the highest degree of immersion [21,22].

Participation motivation can be divided into goal-oriented, activity-oriented, and learning-oriented types depending on the desired direction, and each type has different characteristics. Regarding the goal-oriented type, it refers to training based on a clear goal, such as acquiring skills or qualifications above a certain level. It reflects a relatively fast engagement in active participation and speed of acquisition; however, the level of participation may decrease after the goal is achieved [23]. Regarding the activity- and learning-oriented types, significance is placed on participating in the activity and acquiring knowledge through it, rather than on a specific goal. Participation motivation and training intensity are somewhat weaker than the goal-oriented type. However, since the satisfaction experienced with the act itself is greater, the degree of continuous participation and enjoyment can be high.

The difference between the participation degree and acquisition level can vary depending on participants’ motivation; therefore, participation motivation is an important factor in understanding the overall knowledge or skill-acquisition process [24,25,26]. The higher the participation motivation, the higher the satisfaction, which is directly related to confidence [15].

#### 1.2.2. Leisure Satisfaction

Traditionally, leisure has been centered on travel, recreation, and hobbies. However, there has been a recent increase in the number of individuals enjoying various cultural and leisure experiences that emphasize sensibility and individuality as leisure activities, moving away from previously enjoyed leisure activities. Leisure satisfaction psychologically relieves stress and tension in everyday life, and satisfaction with leisure accompanied by interaction with others or physical activities such as sports can be linked to physical health and confidence in interpersonal relationships [27]. Leisure satisfaction is subjective and has a significant psychological component.

Leisure satisfaction can vary depending on the purpose and motivation for participating in leisure activities. Regarding sports or leisure activities that involve participation due to intrinsic motivation and physical activity, the sense of accomplishment and satisfaction can be perceived as relatively high compared to static leisure activities accompanied by rest [16]. Leisure time has also been investigated as a factor affecting satisfaction. The results of a study by Gelissen [28] indicate that increased time spent on leisure activities increases the level of satisfaction. Since leisure is an activity based on individual preferences, sufficient time for its utilization can have a positive effect on satisfaction. Additionally, the higher the frequency of participation, the higher the leisure satisfaction, and the level of satisfaction is an indicator of whether to continue engaging in leisure activities [29]. When leisure satisfaction is high, individuals actively participate in leisure activities, which affects their self-efficacy and increases happiness [30]. As a result, their quality of life increases, ultimately leading to life satisfaction through leisure satisfaction [31].

#### 1.2.3. Self-Efficacy

Self-efficacy is defined as the confidence one feels in performing a specific action such as work or hobbies, and the belief that one can properly perform a specific action or attain a specific goal [32]. Physical self-efficacy is a positive and strong belief in one’s abilities in sports or physical activities. Self-efficacy contributes to determining behavior [33]. When self-efficacy is high, the ability to perform an action or task is also high, and individuals make continuous efforts to increase their abilities; therefore, the degree of performance and self-efficacy may be proportional. Self-efficacy is a major factor explaining goals, actions, and results.

High physical self-efficacy is believed to positively affect overall sports behavior, suggesting a strong sense of challenge and the ability to make effective decisions in any situation [34]. Self-efficacy in a specific area affects behavior. In sports, physical self-efficacy can act as a factor leading to continued motivation and positive performance [35]. Previous studies confirmed that self-efficacy significantly influences performance [36]. Self-efficacy affects attitude; when self-efficacy is high, individuals are more proactive and have a higher interest than when it is low, which can lead to better performance [37]. Therefore, self-efficacy plays an important role in achieving the desired results.

### 1.3. Setting of Hypotheses

**Figure 1:**
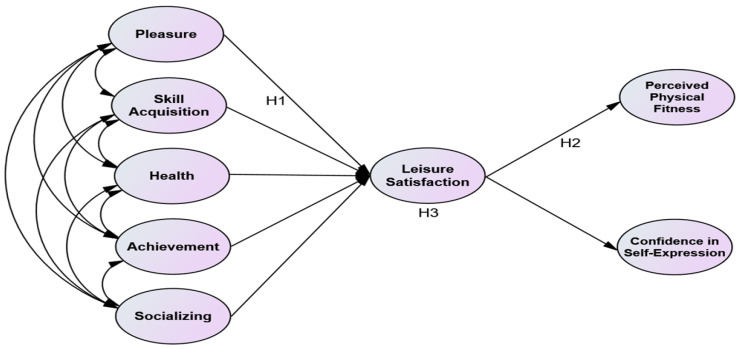
Research model.

This study examined the effects of participation motivation in sports climbing on leisure satisfaction and physical self-efficacy. To achieve the purpose of this study, the following hypotheses were formulated based on a literature review, and a research model was presented. Figure 1 illustrates the research model.

#### 1.3.1. Effect of Participation Motivation in Sports Climbing on Leisure Satisfaction

Positive aspects of sports climbing, such as strengthening physical muscles, improving endurance, and overcoming mental limitations via high-intensity physical activity, have been documented [6,7,26]. However, in comparison with other leisure activities, sports climbing comprises difficult elements (e.g., risk factors associated with the nature of rock climbing and the physical strength and practice required to reach a certain level of skill) [8,26]. Considering the characteristics of sports climbing and the purpose of participation in sports leisure presented in previous studies [24,25,26], this study divided participation motivation in sports climbing into five factors: pleasure, skill acquisition, health, achievement, and socializing.

**Hypothesis** **1.** *Participation motivation in sports climbing (pleasure, skill acquisition, health, achievement, socializing) has a positive effect on leisure satisfaction.*

#### 1.3.2. The Impact of Leisure Satisfaction on Physical Self-Efficacy

Leisure satisfaction is a positive emotion resulting from leisure activities [38]. Previous studies have suggested that satisfaction obtained from interactions with others, such as sports or leisure activities accompanied by physical activity, can be linked to physical health and confidence in interpersonal relationships [27]. Self-efficacy increases confidence and work effectiveness in daily life and is an important factor that ultimately affects quality of life [33]. The following hypothesis was presented based on the findings of previous studies:

**Hypothesis** **2.** *Leisure satisfaction has a positive effect on physical self-efficacy (perceived physical fitness, confidence in self-expression).*

#### 1.3.3. Verification of the Mediating Effect of Leisure Satisfaction in the Relationship between Participation Motivation in Sports Climbing and Physical Self-Efficacy

Satisfaction obtained from interactions with others, such as sports or leisure activities accompanied by physical activity, can be linked to physical health and confidence in interpersonal relationships [27]. Self-efficacy represents a strong sense of self-confidence and a factor that influences the achievement of desired results [36]. Based on the findings of previous studies, the following hypothesis was established to verify the mediating effect of leisure satisfaction on the relationship between participation motivation in sports climbing and physical self-efficacy:

**Hypothesis** **3.** *Leisure satisfaction mediates the relationship between participation motivation in sports climbing (pleasure, skill acquisition, health, achievement, socializing) and physical self-efficacy (perceived physical fitness, confidence in self-expression).*

## 2. Methods

### 2.1. General Characteristics of Participants

A survey was conducted for approximately five months from early February to the end of June 2023. The participants were adult men and women over 20 years of age who had registered and participated in an indoor sports climbing gymnasium for more than three months. The survey was conducted with the cooperation of five indoor sports climbing gyms operating in Seoul, Gyeonggi province. The survey was conducted using a self-administration method with 350 participants. The survey in the study was voluntary and anonymous. All participants gave consent to participate on the first page of the survey. This study was conducted in accordance with the Bioethics and Safety Act (Ministry of Health and Welfare) (Enforcement 2021. 12. 30.; Ministry of Health and Welfare Ordinance No. 852, 2021. 12. 30., partially amended) Article 13 (research on human subjects exempt from deliberation by Institutional Review Board). The general characteristics of the participants for this study are presented in Table 1.

### 2.2. Operational Definition of Variables and Composition of Measurement Items

#### 2.2.1. Participation Motivation in Sports Climbing

In this study, participation motivation in sports climbing was defined as the purpose or reason for continuing to participate in sports climbing for leisure. Among the motivations for participation, pleasure refers to the fun and joy experienced while participating in exercise, skill acquisition refers to acquiring specialized exercise methods, and health refers to maintaining a good physical and mental condition. Achievement refers to the excitement and satisfaction experienced when completing a difficult skill or program successfully, while socializing is defined as the purpose of interacting with individuals with the same hobbies.

The measurement items, including pleasure, skill acquisition, health, achievement, and socializing, were composed of four questions each, based on related studies such as Pelletier et al. [24], Weissinger and Banalos [25], and Rhim and Kim [26].

#### 2.2.2. Leisure Satisfaction

This study defined leisure satisfaction as the positive emotions gained while engaging in leisure activities. The measurement items comprised seven questions, based on related studies such as Beard and Ragheb [38], Park et al. [29], and Brown and Frankel [31].

#### 2.2.3. Physical Self-Efficacy

This study defined physical self-efficacy as the belief and confidence in one’s physical abilities gained through physical activity. Perceived physical fitness was defined as confidence in having expertise in one’s motor skills, whereas confidence in self-expression was defined as confidence in one’s external appearance or physical strength formed through exercise.

The measurement items comprised six questions for each of the two factors of perceived physical fitness and confidence in self-expression, based on related studies such as Bandura [34], Jang [32], and Gist and Mitchell [33].

### 2.3. Data Collection and Analysis Methods

A total of 350 participants were surveyed, and 324 responses were used for analyses after excluding 26 responses due to missing confirmation results or insincere responses. All survey questions, except those on demographic characteristics, were measured using a 5-point Likert scale. Using AMOS 22 and SPSS 22, we conducted Frequency Analysis, Confirmatory Factor Analysis (CFA) to analyze the validity of the survey tool, Reliability Analysis, and Model Suitability Measurement. Conceptual reliability and construct validity were verified through Correlation Analysis, and finally, the hypotheses were verified through Path Analysis.

## 3. Results

### 3.1. Factor and Reliability Analyses

Confirmatory Factor Analysis (CFA) was conducted to verify the validity and suitability of the variables. The Root Mean Square Residual (RMR) and Root Mean Square Error of Approximation (RMSEA) were less than 1.0, and Goodness of Fit Index (GFI), Adjusted Goodness of Fit Index (AGFI), Comparative Fit Index (CFI), and Normed Fit Index (NFI) values were all greater than 0.7 (between 0.8 and 0.9), confirming a high degree of validity [39,40].

#### 3.1.1. Confirmatory Factor Analysis and Reliability Analysis Results of Participation Motivation in Sports Climbing

Table 2 presents the results of the validity and reliability analyses of participation motivation in sports climbing.

#### 3.1.2. Confirmatory Factor Analysis and Reliability Analysis Results of Leisure Satisfaction

Based on the CFA results, two questions that were unsuitable for analysis since their factor loadings were less than 0.5 were removed and measured, and all values were confirmed to be appropriate. Table 3 presents the results of the validity and reliability analyses for leisure satisfaction.

#### 3.1.3. Confirmatory Factor Analysis and Reliability Analysis Results of Physical Self-Efficacy

Based on the first factor analysis results, two questions regarding confidence in self-expression were confirmed to have factor loadings of less than 0.5. After removal and reanalysis, all values were confirmed to be appropriate. Table 4 presents the results of the validity and reliability analyses for physical self-efficacy.

### 3.2. Verification of Suitability and Validity of the Measurement Model

Measurement model analysis was conducted to verify the one-dimensionality and suitability of the variables extracted through CFA. By setting the covariance between all factors, the analysis results were χ^2^ = 1302.217, df = 532, *p* = 0.000, CMIN/df = 2.448, GFI = 0.819, AGFI = 0.750, CFI = 0.821, NFI = 0.803, RMR = 0.083, and RMSEA = 0.095. All acceptable values were derived to ensure the suitability of the model. Conceptual Reliability and Average Variance Extracted (AVE) values were derived to verify the construct validity. All conceptual reliability values were over 0.7, and AVE values were over 0.5, confirming convergent validity. Discriminant validity was confirmed as the AVE value was higher than the squared correlation coefficient of all factors [39,40]. Since the suitability and validity of the measurement model were ensured, the model was confirmed to be appropriate for hypothesis testing. The results of the correlation analysis are presented in Table 5.

### 3.3. Verification of Hypotheses

#### 3.3.1. Verification Results of Hypotheses 1 and 2

**Figure 2:**
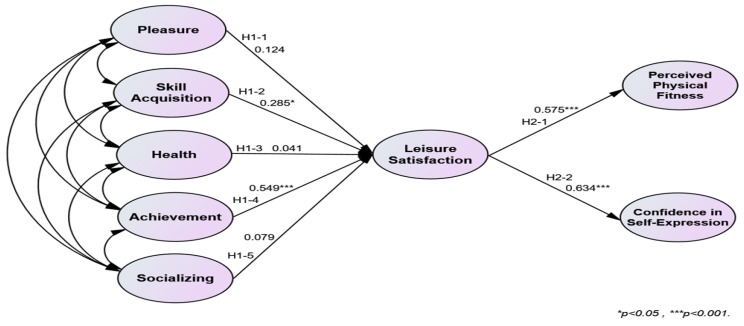
Validated model of Hypotheses 1 and 2.

The verification of Hypothesis 1 was partially accepted. Among the subfactors of participation motivation in sports climbing, only skill acquisition and achievement positively affected leisure satisfaction. The verification of Hypothesis 2 was accepted. Leisure satisfaction positively affected physical self-efficacy. The verification results of Hypotheses 1–2 are presented in Table 6. Figure 2 illustrates the validated model of Hypotheses 1–2.

#### 3.3.2. Verification Results of Hypothesis 3

**Figure 3:**
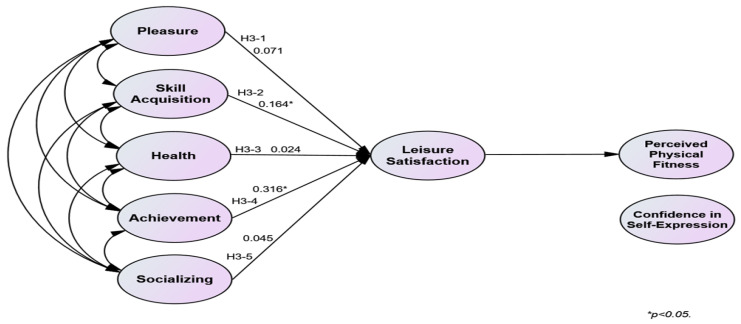
Validated model of Hypotheses 3-1 to 3-5.

**Figure 4:**
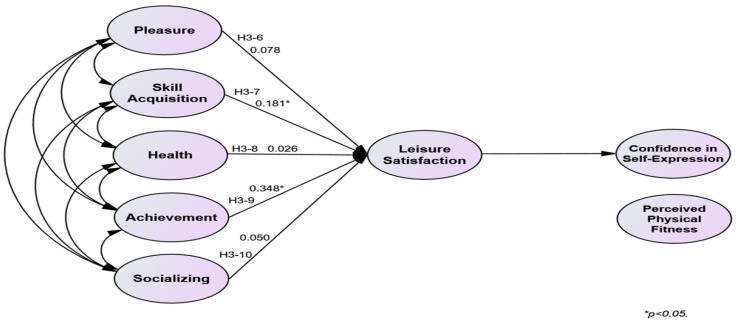
Validated model of Hypotheses 3-6 to 3-10.

The verification of Hypothesis 3 was partially accepted. Leisure satisfaction had a mediating effect when skill acquisition and achievement, among the subfactors of participation motivation, increased in the relationship between participation motivation in sports climbing and physical self-efficacy. The verification results of Hypothesis 3 are present in Table 7. Figure 3 and Figure 4 illustrate the validated model of Hypothesis 3.

## 4. Discussion

This study aimed to determine the impact of participation motivation of amateur sports climbing participants on leisure satisfaction and physical self-efficacy. Based on the results presented in Section 3, the interpretation of the results and empirical application methods are discussed through a comparative analysis with previous studies as follows.

First, Hypothesis 1, “Participation motivation in sports climbing (pleasure, skill acquisition, health, achievement, and socializing) has a positive effect on leisure satisfaction,” was partially accepted. Among the subfactors of participation motivation in sports climbing, only skill acquisition and achievement positively affected leisure satisfaction, while pleasure, health, and socializing did not appear to affect leisure satisfaction. Boyd [41] reported that concerning extreme sports such as skateboarding, motivational factors such as technological development, a sense of accomplishment, and stimulating experiences are highly prevalent motivations for participation, supporting the results of this study. Campbell et al. [42] suggested that specialized pre-participation evaluation is essential due to the exercise specificity of sports climbing. Previous studies indicated that sports climbing differs from general leisure participation motivation owing to the inherent risk of the nature of the exercise, which corresponds with the results of this study. Additionally, the reason why the results of the above-mentioned previous studies and the results of this study appear similar may be due to the difficulty of acquiring the skills for sports climbing and since it has the characteristics of an extreme sport that involves risk in participation. Choi et al. [43] conducted a study on participation motivation, leisure immersion, and satisfaction of golf participants, and the main motivations for participation were pleasure, healing, and socialization. In addition, a study by Koivula [44] analyzed the factors that lead to general participation and found that health, pleasure, ability improvement, and competition were presented as the main motivations for participation. The participation time and degree of participation immersion varied depending on the participation motivation. A comprehensive analysis of the contents of these previous studies indicated that participation motivation significantly varies depending on the type of sport and according to the specificity of sports climbing. The results of this study indicated that skill acquisition and achievement were the main factors affecting leisure satisfaction.

Second, Hypothesis 2, “Leisure satisfaction has a positive effect on physical self-efficacy (perceived physical movement and confidence in self-expression),” was accepted. Hypothesis 2 was established to determine the relationship between leisure satisfaction and physical self-efficacy before verifying the mediating effect of leisure satisfaction on the relationship between participation motivation and physical self-efficacy in sports climbing. Leisure satisfaction positively affected both perceived physical movement and self-expression confidence, which are subfactors of physical self-efficacy. These results indicated that leisure satisfaction through sports climbing positively affected perceived physical movement and confidence and that participants’ leisure satisfaction was a significant factor. Ahn and Hwang [45] revealed that leisure satisfaction has a positive relationship with self-efficacy when participating in leisure sports, while Bum et al. [46] found that participation in leisure activities had a positive effect on self-efficacy and social adaptation. The results of the aforementioned studies support those of this study.

Third, Hypothesis 3, “Leisure satisfaction mediates the relationship between participation motivation in sports climbing (pleasure, skill acquisition, health, achievement, and socializing) and physical self-efficacy (perceived physical movement and confidence in self-expression),” was partially accepted. In the influence relationship between participation motivation in sports climbing and perceived physical movement among sub-factors of physical self-efficacy, leisure satisfaction was found to have a mediating effect when skill acquisition and achievement, among the participation motivation sub-factors, increased. Additionally, leisure satisfaction had a mediating effect when skill acquisition and achievement, among the subfactors of participation motivation, increased, among the subfactors of physical self-efficacy, in the relationship between participation motivation in sports climbing and self-expression confidence. Consequently, it is defined as a sport that is difficult to access owing to its high risk and is presented as having a high demand for the sense of accomplishment associated with the sport and the acquisition of specialized skills. As previously mentioned, the characteristic of tending to have a strong sense of accomplishment and motivation to acquire professional skills is consistent with the main motivation for participation in extreme sports presented by Boyd [41], which is consistent with the results of this study. Campbell et al. [42] mentioned that participation motivation in general leisure activities and sports climbing differs according to the exercise specificity of sports climbing. Considering the overall contents of previous studies, sports climbing has similar characteristics to extreme sports, with a high level of difficulty. The present study produced similar results to those of Campbell et al. [42]. However, a recent study by Choi et al. [43] suggested that the main motivations for participation in golf are pleasure, healing, and socializing, and participation motivation significantly varies depending on the characteristics of the sport. Additionally, Koivula [44] emphasized that participation attitudes vary depending on participation motivation in sports, whereas Brymer and Schweitzer [47] noted that few studies have been conducted on participation motivation in extreme sports. In a situation where little research on sports climbing has been conducted, this study not only identified the characteristics of sports climbing and its specific motivations but also set leisure satisfaction as a mediating variable in the relationship between participation motivation in sports climbing and physical self-efficacy. Therefore, this study attempted to comprehensively analyze the causal relationships among the variables. This study found that when skill acquisition and achievement increased among the sub-factors of participation motivation in sports climbing, it not only had a positive effect on leisure satisfaction but also positively affected physical self-efficacy. Specifically, when interpreting these results, the improvement in leisure satisfaction through participation in sports climbing suggests that it increases physical self-efficacy, which includes improvements in physical abilities such as skills, physical strength, physique, agility, and situational response as perceived by the participants, as well as confidence in interpersonal relationships. However, as previously mentioned, to induce continuous participation, it is important to understand the characteristics of sports climbing and the main motivations for participation. Considering the characteristics of sports climbing based on the results of this study, it is a type of sport in which it is difficult to acquire skills, and it requires individuals to take risks, desire new adventures, and be highly motivated to participate in skill acquisition and achievement. Therefore, continuous research on sports characteristics is necessary to ensure continued participation in sports climbing.

## 5. Conclusions

### 5.1. Empirical Implications

This study aimed to verify the effects of participation motivation in sports climbing on leisure satisfaction and physical self-efficacy. To comprehensively verify these details, this study set leisure satisfaction as a mediating variable in the influence relationship between participation motivation in sports climbing and physical self-efficacy and attempted to analyze the causal relationship between the variables. Furthermore, sports climbing has received significant attention since its adoption as an official sport at the 2020 Olympics, and the number of participants is rapidly increasing. However, only a few related studies have been conducted. Therefore, the characteristics of sports climbing are not well established and systematic education that matches participation motivation has not been properly provided. The results of this study present empirical implications that can be applied in the field of sports climbing instruction.

First, through a comparative analysis of the results of this study and previous studies, sports climbing can be classified as an extreme sports category that involves a sense of adventure and risk-taking. Therefore, this study suggests that a pre-participation evaluation is necessary before participating in sports climbing and that a standardized counseling manual that reflects the unique characteristics of sports climbing is needed.

Second, the results of this study indicated that among participation motivation in sports climbing, skill acquisition and achievement were the main motivations for participation and positively affected leisure satisfaction and physical self-efficacy. Thus, skill acquisition is a significant factor for sports climbing participants, and it is necessary to establish a systematic system for sports climbing instruction and educational programs. For example, if a system similar to Taekwondo’s promotional system is established, participants can understand their current levels and set future goals. In addition, promotion to a higher level based on skill acquisition can increase participants’ achievement and motivate them to continue exercising. If this promotion system is developed into an official qualification examination system under the supervision of sports climbing-related associations, the number of loyal participants in sports climbing may continue to increase.

Third, since sports climbing requires a sense of adventure, and due to the risks associated with participation, it is important to convey awareness to participants, ensuring their safe participation. This study suggests that continuous safety inspections of facilities and a safety certification system should be established to ensure that participants can participate safely, while continuous safety education for coaches should be provided by government sports-related organizations.

### 5.2. Proposals

Through a comprehensive analysis of the causal relationship between the variables established during this study, meaningful results were derived from the exercise characteristics of sports climbing, and the relationship between the main participation motivations and physical self-efficacy was identified. However, since few studies have been conducted on sports climbing, several follow-up studies are required in the future and the following suggestions may guide the direction and content of future studies.

First, this study was limited to quantitative research to verify the effects of participation motivation on leisure satisfaction and physical self-efficacy. However, various research methods need to be used to conduct a detailed and specific verification of each variable. Therefore, follow-up studies should consider using mixed-method designs, such as qualitative research using interviews with participants or conducting comparative analysis by conducting qualitative and quantitative analyses simultaneously.

Second, sports climbing was found to have the same main participation motivation as extreme sports, characterized by a sense of adventure and the need to take risks due to participation. Thus, future studies should compare the characteristics of different sports, such as sports climbing, extreme sports, and general leisure sports.

Third, participation in leisure sports is important since it can ultimately improve one’s quality of life. Therefore, this study suggests that it is necessary to conduct research by subdividing participants by sex, age, occupation, etc., to expand and sustain participation in leisure sports in follow-up studies.

## Figures and Tables

**Table 1:** Research participants’ general characteristics.

Division	Factors	Frequency	Percentage (%)	Division	Factors	Frequency	Percentage (%)
Gender	Male	237	73.1	Occupation	Student	62	19.1
	Female	87	26.9		Housewife	12	3.7
Age	20’s	87	26.9		Office worker	129	39.8
	30’s	123	37.9		Self-employed	74	22.9
	40’s	63	19.4				
	50’s	32	9.9		Professional worker	43	13.3
	over 60	19	5.9		Others	4	1.2
Experience	Less than 1 year	98	30.2	Frequency /Week	1–2 times	145	44.8
	1 year to less than 3 years	121	37.3		3–4 times	111	34.3
	3 years to less than 5 years	64	19.8		5–6 times	40	12.3
	5 years or more	41	12.7		7 times	28	8.6

**Table 2:** Validity and reliability analyses for participation motivation in sports climbing.

Factors	Items		Standardized Coefficient	S.E.	C.R.	*p*	Reliability
Participation motivation in sports climbing	Pleasure	Sports climbing is fun.	0.765				0.847
		I experience pleasure when I do sports climbing.	0.867	0.103	11.517	***	
		I like the feeling of being completely focused when I do sports climbing.	0.888	0.100	11.517	***	
		Participating in sports climbing is always fun.	0.613	0.131	7.770	***	
	Skill acquisition	It is fun to learn the various movements of sports climbing.	0.654				0.849
		Learning new exercise methods is fun.	0.852	0.163	8.538	***	
		My skills and physical strength are gradually improving.	0.706	0.166	7.512	***	
		I want to continue learning high-level skills.	0.548	0.182	6.991	***	
	Health	My condition improves when I do sports climbing.	0.742				0.860
		I like the refreshing feeling after exercising.	0.703	0.102	8.700	***	
		Sports climbing helps with weight control.	0.831	0.098	10.390	***	
		Sports climbing gives me confidence in everything I do.	0.795	0.109	9.917	***	
	Achievement	I feel good when I succeed at a difficult level.	0.562				0.833
		I want to succeed on a difficult level.	0.893	0.173	7.658	***	
		I want to challenge myself when given a difficult task.	0.889	0.179	7.644	***	
		I want to go to the next level quickly.	0.801	0.199	7.257	***	
	Socializing	I can make friends while doing sports climbing.	0.645				0.834
		Participating in sports climbing allows me to get along comfortably with others.	0.879	0.167	9.101	***	
		Participating in sports climbing allows me to spend time with nice people.	0.910	0.161	9.269	***	
		I can meet up with people after sports climbing.	0.712	0.165	7.765	***	
χ^2^ = 307.296, df = 160, *p* = 0.000, CMIN/df = 1.921, GFI = 0.848, AGFI = 0.800, CFI = 0.924, NFI = 0.855, RMR = 0.036, RMSEA = 0.076							

*** *p* < 0.001.

**Table 3:** The validity and reliability analyses for leisure satisfaction.

Factor	Items	Standardized Coefficient	S.E.	C.R.	*p*	Reliability
Leisure Satisfaction	I like being able to try new things.	0.667				0.817
	I like being able to gain knowledge about exercise.	0.643	0.244	5.979	***	
	It allows me to improve my athletic ability.	0.821	0.206	6.497	***	
	It is good to meet people with similar hobbies.	0.846	0.176	6.621	***	
	It helps improve health.	0.866	0.210	6.037	***	
χ^2^ = 137.079, df = 84, *p* = 0.000, CMIN/df = 1.631, GFI = 0.824, AGFI = 0.752, CFI = 0.812, NFI = 0.805, RMR = 0.063, RMSEA = 0.091						

*** *p* < 0.001.

**Table 4:** The validity and reliability analyses for physical self-efficacy.

Factor	Items		Standardized Coefficient	S.E.	C.R.	*p*	Reliability
Physical Self-Efficacy	Perceived Physical Fitness	I have excellent reflexes.	0.532				0.904
		I act quickly.	0.661	0.236	6.032	***	
		I have good physical strength.	0.824	0.261	6.760	***	
		I have a body suitable for sports climbing.	0.883	0.221	6.927	***	
		I am good at handling situations.	0.526	0.169	6.999	***	
		I am evaluated as being technically excellent.	0.656	0.237	6.002	***	
	Confidence in Self-Expression	I can speak confidently about my experience.	0.525				0.849
		I want to teach others how to exercise.	0.700	0.229	6.255	***	
		I can exercise well.	0.937	0.248	6.173	***	
		My body shape was trained through exercise.	0.695	0.221	6.343	***	
χ^2^ = 132.217, df = 34, *p* = 0.000, CMIN/df = 3.889, GFI = 0.853, AGFI = 0.772, CFI = 0.853, NFI = 0.815, RMR = 0.081, RMSEA = 0.085							

*** *p* < 0.001.

**Table 5:** Correlation analysis results.

Factors	1	2	3	4	5	6	7	8
1	1							
2	0.447	1						
3	0.505	0.491	1					
4	0.273	0.511	0.331	1				
5	0.426	0.310	0.487	0.189	1			
6	0.463	0.462	0.532	0.448	0.351	1		
7	0.138	0.289	0.167	0.291	0.076	0.308	1	
8	0.227	0.389	0.263	0.312	0.111	0.358	0.192	1
Construct Reliability	0.936	0.786	0.916	0.923	0.929	0.843	0.862	0.881
AVE	0.788	0.647	0.733	0.755	0.771	0.592	0.522	0.666

The square of each correlation coefficient was calculated. 1. Pleasure; 2. Skill Acquisition; 3. Health; 4. Achievement; 5. Socializing; 6. Leisure Satisfaction; 7. Perceived Physical Fitness; 8. Confidence in Self-Expression.

**Table 6:** The verification results of Hypotheses 1 and 2.

Hypothesis	Path	Standardized Coefficient	S.E	C.R	*p*	Results
1-1	Pleasure → Leisure Satisfaction	0.124	0.099	1.166	0.244	Rejected
1-2	Skill Acquisition → Leisure Satisfaction	0.285	0.127	2.115	0.034 *	Accepted
1-3	Health → Leisure Satisfaction	0.041	0.112	0.281	0.779	Rejected
1-4	Achievement → Leisure Satisfaction	0.549	0.142	3.670	***	Accepted
1-5	Socializing → Leisure Satisfaction	0.079	0.072	1.104	0.270	Rejected
2-1	Leisure Satisfaction → Perceived Physical Fitness	0.575	0.179	3.666	***	Accepted
2-2	Leisure Satisfaction → Confidence in Self-Expression	0.634	0.169	3.499	***	Accepted

* *p* < 0.05, *** *p* < 0.001.

**Table 7:** Hypothesis 3 verification results.

Hypothesis	Path	Standardized Coefficient	Indirect Coefficient		*p*	Results
			Min	Max		
3-1	Pleasure → Leisure Satisfaction → Perceived Physical Fitness	0.071	−0.107	0.265	0.255	Rejected
3-2	Skill Acquisition → Leisure Satisfaction → Perceived Physical Fitness	0.164	0.005	0.470	0.047 *	Accepted
3-3	Health → Leisure Satisfaction → Perceived Physical Fitness	0.024	−0.327	0.262	0.776	Rejected
3-4	Achievement → Leisure Satisfaction → Perceived Physical Fitness	0.316	0.178	0.500	0.003 *	Accepted
3-5	Socializing → Leisure Satisfaction → Perceived Physical Fitness	0.045	−0.042	0.160	0.261	Rejected
3-6	Pleasure → Leisure Satisfaction → Confidence in Self-Expression	0.078	−0.118	0.255	0.251	Rejected
3-7	Skill Acquisition → Leisure Satisfaction → Confidence in Self-Expression	0.181	0.010	0.468	0.050 *	Accepted
3-8	Health → Leisure Satisfaction → Confidence in Self-Expression	0.026	−0.324	0.266	0.798	Rejected
3-9	Achievement → Leisure Satisfaction → Confidence in Self-Expression	0.348	0.209	0.529	0.003 *	Accepted
3-10	Socializing → Leisure Satisfaction → Confidence in Self-Expression	0.050	−0.053	0.163	0.278	Rejected

* *p* < 0.05.

## Data Availability

The data that support the findings of this study are available from the corresponding author upon reasonable request.
